# A Multiscale, Mechanism-Driven, Dynamic Model for the Effects of 5α-Reductase Inhibition on Prostate Maintenance

**DOI:** 10.1371/journal.pone.0044359

**Published:** 2012-09-06

**Authors:** Michael G. Zager, Hugh A. Barton

**Affiliations:** 1 Pharmacokinetics, Dynamics and Metabolism, Worldwide Research and Development, Pfizer, Inc., San Diego, California, United States of America; 2 Pharmacokinetics, Dynamics and Metabolism, Worldwide Research and Development, Pfizer, Inc., Groton, Connecticut, United States of America; The Chinese University of Hong Kong, Hong Kong

## Abstract

A systems-level mathematical model is presented that describes the effects of inhibiting the enzyme 5α-reductase (5aR) on the ventral prostate of the adult male rat under chronic administration of the 5aR inhibitor, finasteride. 5aR is essential for androgen regulation in males, both in normal conditions and disease states. The hormone kinetics and downstream effects on reproductive organs associated with perturbing androgen regulation are complex and not necessarily intuitive. Inhibition of 5aR decreases the metabolism of testosterone (T) to the potent androgen 5α-dihydrotestosterone (DHT). This results in decreased cell proliferation, fluid production and 5aR expression as well as increased apoptosis in the ventral prostate. These regulatory changes collectively result in decreased prostate size and function, which can be beneficial to men suffering from benign prostatic hyperplasia (BPH) and could play a role in prostate cancer. There are two distinct isoforms of 5aR in male humans and rats, and thus developing a 5aR inhibitor is a challenging pursuit. Several inhibitors are on the market for treatment of BPH, including finasteride and dutasteride. In this effort, comparisons of simulated vs. experimental T and DHT levels and prostate size are depicted, demonstrating the model accurately described an approximate 77% decrease in prostate size and nearly complete depletion of prostatic DHT following 21 days of daily finasteride dosing in rats. This implies T alone is not capable of maintaining a normal prostate size. Further model analysis suggests the possibility of alternative dosing strategies resulting in similar or greater effects on prostate size, due to complex kinetics between T, DHT and gene occupancy. With appropriate scaling and parameterization for humans, this model provides a multiscale modeling platform for drug discovery teams to test and generate hypotheses about drugging strategies for indications like BPH and prostate cancer, such as compound binding properties, dosing regimens, and target validation.

## Introduction

With the availability of information describing many individual components of biological systems, there is increasing focus on developing multiscale computational models that capture the overall systems behavior [1]. Many systems biology models address complex intracellular signaling pathways, while other efforts focus on the function of individual organs such as the heart [2], [3]. The challenge of modeling endocrine function is that the system involves multiple organs (those secreting hormones into blood and those responding), responses that progress from changes in gene expression through to changes in cellular and organ function, and frequently multiple feedback processes such that decisions around the level of biological detail to incorporate are challenging [4], [5]. The intended use for a biological model is typically a major driving force for decisions around the needed level of detail. Here the challenge was to incorporate pharmacological interventions into a model based upon surgical manipulation (i.e., castration) to ask about the capabilities of the model as well as obtain perspectives on the drivers for such interventions for purposes of drug discovery.

The enzyme 5α-reductase (5aR) plays a critical role in regulating of the size and function of the ventral prostate. Testosterone (T) is metabolized by 5aR into the more potent 5α-dihydrotestosterone (DHT) [6], [7], the driving force of prostate gene-regulation [8], [9], [10], [11], [12], [13], [14]. In turn, DHT controls the gene expression of 5aR, creating a feedback control loop [15]. There are two known isoforms of 5aR in rats and humans [6], [7], [16], [17], [18], [19]. One isoform (labeled 5aR1 in this article) is widely distributed in the body and is expressed abundantly in the liver of rats, a major tissue for T metabolism. The other isoform (labeled 5aR2 in this article) has been reported to be expressed mainly in androgen-dependent tissues and is abundant in the prostate of rats, the other major site for T metabolism [6]. However, there exists at least one reference reporting nearly equal distribution of 5aR1 and 5aR2 in prostate tissue of rats [19]. In human cancerous prostate tissue, expression levels for each isoform have been shown to increase [20], [21].

The exact nature of the binding exhibited by each of the two predominant 5aR inhibitors on the market, finasteride and dutasteride, for each isoform has not always been clear. Prior to the discovery of a second 5aR isoform, finasteride was believed to be a selective, competitive, reversible inhibitor of 5aR2 [22]. But a phase I study showing a 7-day requirement for DHT levels to return to baseline after nearly 80% depletion following finasteride dosing demonstrated the possibility of a more complex mechanism, given the half-life of finasteride is approximately 6–8 hours in humans [22], [23]. With the recognition of a second 5aR isoform, it was thought that finasteride was a time-dependent inhibitor of both 5aR1 and 5aR2 [22]. Time dependent inhibition results from an enzyme-inhibitor bond with a very long half life (often on the order of many days), rendering the enzyme effectively useless. Finally, finasteride was shown to be a weakly competitive, reversible inhibitor of 5aR1 and a potent, time-dependent inhibitor of 5aR2 [21], [24].

A new class of compounds was subsequently developed, including the first marketed, potent, dual 5aR inhibitor, dutasteride [22]. Dutasteride is considered to be a potentially desirable alternative to finasteride, due to its longer half life and greater potency against each isoform [21], [25], [26]. It was suggested by Stuart and coworkers, however, that both finasteride and dutasteride exhibit competitive, reversible inhibition on 5aR1 and time-dependent inhibition on 5aR2 [26], albeit with different potencies against each isoform.

Since approval of finasteride and dutasteride for treatment of benign prostatic hyperplasia (BPH), data abound characterizing 5aR inhibitors in prostate cancer prevention and treatment [12], [22], [27], [28], [29], [30], [31], [32]. Even in androgen-independent (hormone refractory) prostate cancer, it is believed androgens play a significant role in tumor growth [33].

Classical compartmental pharmacokinetic (PK) models for both dutasteride and finasteride have been published [22], [25], [26]. Furthermore, there have been recent advances in developing more mechanistic models for 5aR inhibition by finasteride and subsequent effects on the prostate [34], [35]. However, the authors are unaware of any mechanistically-based PK or pharmacodynamic modeling efforts to date that account for the complex binding kinetics of finasteride to 5aR1 and5aR2 and associate 5aR inhibition with downstream gene regulartory-driven changes in prostate mass. Approaches such as this can be adapted to aid in quantitatively understanding the different mechanisms of inhibition exhibited by these compounds along with the nonintuitive, inherent behavior of the hormonal regulatory system associated with prostate maintenance. These understandings can yield better proposed dosing regimens in clinical trials as well as aiding in chemistry design strategies in drug discovery.

The objective of this research effort was to develop a systems-level mathematical model to simulate the different mechanisms of inhibition exhibited by finasteride and their gene-regulatory effects on the maintenance of the ventral prostate in adult male rats. To accomplish this objective, a model previously developed for the simulation of prostate maintenance in adult intact and castrated male rats was used as a base model (called the PM model in this article) and suitably augmented to simulate the complexities of 5aR inhibition by finasteride and associated prostatic effects [36].

PM describes the endogenous hormone kinetics of the testicular-pituitary axis and the dynamics of the androgenic regulation of the prostate. The model includes the pharmacokinetics of T, DHT and luteinizing hormone (LH), as well as the dynamics of AR binding and subsequent events leading to the regulation of the prostate. Development of the model included characterization of critical biological and physiological processes inherent in the hormonal regulation of the male reproductive system, including androgen-LH feedback loops and AR-mediated gene transcriptional regulation of four distinct processes: prostatic cell proliferation, anti-apoptosis, fluid production, and 5aR2 activity. While the first three are complex multigene regulatory processes, a simplified description with one set of key regulatory genes for each process was assumed. Although there are two known isoforms of 5aR, only 5aR2 production is explicitly described with a gene in PM, since liver metabolism of T is described using simple *V_max_* and *K_m_* kinetics while prostatic T metabolism includes a more complex description (see [36]).

To simulate prostatic responses to 5aR inhibitor exposurethe model description for 5aR was expanded in both prostate and liver, adding new parameters, some with unknown values. Estimates for most of these values can be obtained from experimental data in the literature. Remaining unknown parameters need to be estimated via model calibration using experimental data for finasteride. Thus, the resulting model is specifically parameterized for finasteride. However, since finasteride and dutasteride were shown to exhibit similar mechanisms of inhibition on the 5aR isoforms, the finasteride model (FM) can be re-parameterized for dutasteride by accounting for differences in dutasteride PK and affinity for each 5aR isoform. This new model, calibrated for rats as a widely used preclinical species with relatively rich data, was used to ask questions important to drug discovery including predicting differential effects of dosing regimens. Translation of such findings to inform clinical studies would require additional model calibration for humans to address the limitations of the rat as a model for prostatic diseases.

## Results

**Figure 1:**
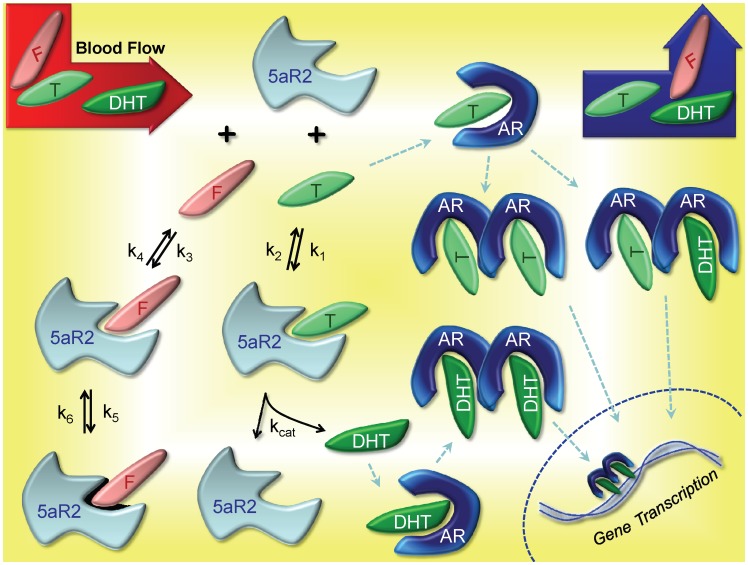
Kinetics of 5aR2-mediated Gene Regulation in the Prostate. Blood flow through the prostate delivers and clears finasteride (F), T and DHT from the compartment. Within the prostate, F competes (*k_3_* and *k_4_*) with T (*k_1_* and *k_2_*) to bind with 5aR2, but F undergoes a further time-dependent inhibition (*k_5_* and *k_6_*) effectively eliminating the enzyme from the active pool. T exhibits typical enzyme-substrate kinetics, where the final product is DHT and the enzyme is released back to the pool of free 5aR2. Free T and DHT bind to AR, subsequently form homo- and heterodimers, and finally bind to any of the four DNA sites of FM for transcription (cell proliferation, anti-apoptosis, fluid production and 5aR2 production (see text and [36]). Although HSP27, HSP90, cofactors and other molecules are involved in the process of chaperoning the dimers into the nucleus and inducing transcription [33], the process is simplified in both PM and FM. For complete details on equations, parameters and figures for dimerization and gene transcription, the reader is referred to [36].

The goal of FM is to simulate gene-regulated prostate dynamics for intact, finasteride-treated and castrated rats. The signaling kinetics leading to androgen-driven gene transcription in the prostate, which ultimately results in regulation of prostate size and function are depicted in Figure 1. In light of finasteride’s different inhibition mechanism for each 5aR isoform, several enhancements of the prior model were required, notably for metabolism of T in the liver and prostate. Finasteride exposure estimates following oral dosing were also needed.

**Figure 2:**
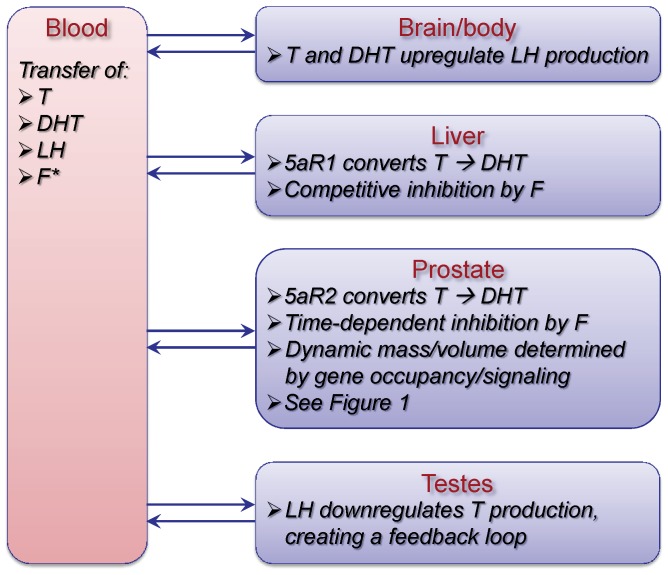
FM – a multiscale, mechanism-driven, dynamic model. FM is comprised of physiological compartments, where critical processes occur such as metabolism, intra- and intercellular signaling, gene transcription and tissue response. Mass transfer of T and DHT takes place between the blood and tissue compartments, while regulatory signaling occurs via AR, LH, T and DHT (also see Figure 1), mainly between the brain, testes and prostate. During intact and finasteride treated simulations, the testicular-pituitary axis forms a negative feedback loop between the testes and the brain via LH, T and DHT. In FM, the brain is lumped into the body compartment, which comprises all tissues and organs not explicitly accounted for in the model structure. The kinetic and gene transcriptional processes involving T, DHT and AR depicted in Figure 1 are fully incorporated in the prostate compartment of FM, resulting in regulation of prostate mass. For further detail, see text and [36]. The kinetics of finasteride (F) in blood are accounted for using a specific pharmacokinetic model developed previously (see Figure 3) and linked directly into FM.

These changes in prostate kinetics and dynamics occur in the broader context of the whole body, including pharmacokinetics of endogenous T and DHT described with physiologically based pharmacokinetic (PBPK) models and androgen-LH signaling between the brain and testes forming the testicular-pituitary axis (Figure 2). Augmented with a significantly more mechansistic description of prostatic events, the overall whole body model structure of FM remains similar to that of PM (see [36]). The changes to the model to incorporate 5aR inhibition are described below along with other improvements made in the modeling for prostate gene occupancy and prostatic cellular mass following which the model calibration is reported. Lastly, we describe the results of simulation studies to investigate the complex interplay of hormonal regulation and kinetics and the kinetics of the inhibitor as influenced by the dosing regimen.

### Building the Finasteride Model (FM)

#### T metabolism in the liver

The liver and prostate are sites of significant T metabolism. 5aR1 is abundantly expressed in the liver while 5aR2 is found at considerably lower levels. Also, 5aR1 is not abundant in the prostate, where 5aR2 is. In light of this, and although 5aR1 is found in other tissues of the rat body, we assume in the model that 5aR1 exists exclusively in the liver and no 5aR2 is expressed. We further assume that metabolism of T by 5aR1 in other tissues is negligible relative to the liver. Finally, it is assumed that 5aR1 in the liver is subject to limited androgen regulation, so its activity is assumed constant under conditions of castration and 5aR inhibitor treatment.

Metabolism of T to DHT via 5aR in the liver was previously modeled with a Michaelis-Menten term, and linear terms were included for the general metabolic clearance of T and DHT in the liver [37]. To represent reversible, competitive inhibition of 5aR1, we added the following standard augmented Michaelis-Menten metabolism term for finasteride-inhibited T metabolism in the liver:
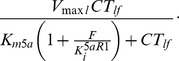



The new equations for T and DHT in the liver are:
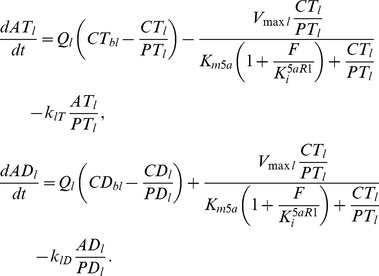
(1)


Descriptions for all state variable and parameter abbreviations in equations are found in Tables 1, 2 and 3. The value for the finasteride inhibition constant *K_i_^5aR1^* is given in [26] as 5.4 nM. The value for the T metabolism *K_m_* (*K_m5a_* in the model) was reported as 40 nM in PM, based on Nnane et al. [12]. Stuart and coworkers reports a value of 2.3 nM, which is the value used in FM [26]. All other parameter values in these equations were left unchanged from PM. The free concentration of finasteride in the liver, *F*, is described in a later section.

**Table 1:** State variables for equations (1) – T metabolism in the liver.

Variable	Description	Units
*AT_l_*	Amount of T in liver	nmol
*AD_l_*	Amount of DHT in liver	nmol
*CT_bl_*	Concentration of T in blood	nM
*CT_l_*	Free concentration of T in liver	nM
*CD_bl_*	Concentration of DHT in blood	nM
*CD_l_*	Free concentration of DHT in liver	nM
*F*	Central compartment free concentration of finasteride^1^	nM

1 New state variable for FM.

#### T metabolism in the prostate

Similar to the assumption that 5aR1 is exclusively found in the liver, we simplify the model with the assumption that 5aR2 is exclusive to the prostate, consistent with Pozzi and coworkers, who reported the only other tissues significantly expressing 5aR2 are androgen-dependent tissues [6]. These remaining tissues likely do not add significant metabolism to the system.

Unlike the simple expression used for classical competitive inhibition in the liver, the model must be augmented for time-dependent inhibition in the prostate. Thus, equations were implemented for the biochemical interactions between T, 5aR2 and finasteride, depicted in Figure 1. These enzyme kinetic equations for FM are given by:
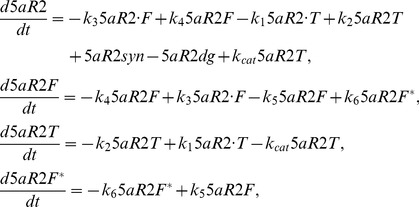
(2)where the enzyme synthesis and degradation terms for 5aR2 are given by: 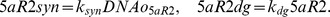



**Table 2:** Parameters for equations (1) – T metabolism in the liver.

Parameter	Description	Units	Value
*Q_l_*	Blood flow rate to liver	L/hr	1.06
*V_maxl_*	Maximum velocity of liver Tmetabolism to DHT	nmol/hr	3.65
*PT_l_*	T-liver partition coefficient	–	2.75
*PD_l_*	DHT-liver partition coefficient	–	2.0
*K_m5a_*	Liver T metabolism K_m_ for 5aR1^1^	nM	2.3
*k_lT_*	Nonspecific liver T elimination	hr^−1^	87.9
*K_lD_*	Nonspecific liver DHT elimination	hr^−1^	77.2
*K_i_^5aR1^*	5aR1 inhibition constant (K_i_)for finasteride^1,2^	nM	5.4

1 Value from [26].

2 New parameter for FM.

Descriptions for all state variable and parameter abbreviations in equations are found in Tables 3 and 4. Similar to the feedback mechanism in PM that controlled prostatic T metabolism via 5aR, the rate of synthesis of 5aR2 in FM is a function of gene occupancy. The parameter *k_dg_* is a fitted parameter. The parameter *k_syn_* is a steady-state forcing parameter that is given by:
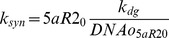
where *5aR2_0_* is the initial (steady state) value for the state variable *5aR2* (the concentration of 5aR2 in the prostate), *DNAo_5aR2_* is the occupancy of the 5aR2 gene (an algebraic value calculated from state variables) and *DNAo_5aR20_* is the initial (steady state) value of *DNAo_5aR2_*.

**Table 3:** State variables for equations (2) –5aR2 kinetics.

Variable	Description	Units
*5aR2*	Free 5aR2 in prostate	nM
*5aR2T*	5aR2:T complex	nM
*5aR2F*	5aR2:finasteride complex	nM
5aR2F*	Permanently bound 5aR2:finasteride	nM
*F*	Concentration of finasteride in central compartment^1^	nM

All new state variables for FM.

1 This algebraic variable is calculated using the finasteride model estimate of finasteride amount (*A2*) in the central compartment along with the molecular weight of finasteride and the estimated volume of distribution of the central compartment for finasteride; see text and Text S3, Table S5.

**Table 4:** Parameters for equations (2) –5aR2 kinetics.

Parameter	Description	Units	Value
*k_1_*	Association rate constant for T:5aR2^1,5^	hr^−1^ nM^−1^	500
*k_2_*	Dissociation rate constant for T:5aR2^1^	hr^−1^	30
*k_cat_*	Catalysis rate constant for T → DHT^1,4^	hr^−1^	270
*K_m_*	K_m_ for T → DHT^3^	nM	0.6
*k_3_*	Association rate constant for finasteride:5aR2^1,5^	hr^−1^ nM^−1^	1000
*k_4_*	Dissociation rate constant for finasteride:5aR2^1^	hr^−1^	500
*k_5_*	Rate constant for *5aR2F* → 5aR2F* 1,3	hr^−1^	3.96
*k_6_*	Rate constant for 5aR2F* → *5aR2F* 1,3	hr^−1^	0
*K_i_^5aR2^*	K_i_ for finasteride:5aR2^1,3^	nM	0.5
*k_dg_*	Degradation rate constant for 5aR2^6^	hr^−1^	0.016
*k_syn_*	Synthesis rate constant for 5aR2	nM/hr	see text
*DNAo_5aR2_*	Prostatic 5aR2 gene occupancy^2^	fraction	variable
*DNAo_5aR20_*	Prostatic 5aR2 gene occupancyinitial value	fraction	0.95

1 New parameter for FM.

2 Algebraic variable; see Text S1.

3 Value from [26].

4 Value from [39].

5 FM not sensitive to this parameter; biologically plausible value chosen (see text).

6 Fitted value; see text.

Since there are now explicit terms in FM for prostatic 5aR2 concentrations (free and bound), an estimate is required for the total concentration at steady-state. To obtain this value we used the estimated 5aR2 concentration in prostate of <0.001% [38], the measured ventral prostate protein of 53±5 µg/g tissue [38], and a molecular weight of 28772 daltons for the 254 amino acid containing 5aR2. The resulting estimate of 18.4 nM was rounded to 20 nM for use in FM due to the uncertainty in the estimate. Thus, at steady-state, all terms that include 5aR2 should total 20 nM.

Figure 1 gives contextual reference to the parameters *k_1_*, *k_2_*, *k_3_*, *k_4_*, *k_5_*, *k_6_* and *k_cat_*. The parameter *k_cat_*, the catalysis term for T metabolism by 5aR2 to DHT, takes the value of 270 hr^−1^ (from [39], reported value of 0.075 sec^−1^). The value for *k_1_* is fitted. We used the established formula from Segal [40], *k_2_ = K_m_k_1_– k_cat_*, to calculate the value of *k_2_*. Values for *K_m_* (prostatic T to DHT metabolism) are variable in the literature –0.6 nM [26], 24.6 nM [39], and 74 [19]. For reasons described later, the Stuart and coworkers value (0.6 nM) was used in FM. With the values of *k_1_*, *K_m_* and *k_cat_* obtained, the value for *k_2_* can then be calculated. The value for *k_3_* is fitted. Stuart and coworkers reports a *K_i_^5aR2^* value for finasteride-5aR2 binding to be 0.5 nM, and therefore we calculated *k_4_* = *K_i_^5aR2^k_3_*. The values for parameters *k_5_* and *k_6_* are reported by Stuart and coworkers to be 3.96 hr^−1^ and approximately zero, respectively. In the model, zero value is used for *k_6_*.

The original equations in PM for T and DHT amounts in the prostate, respectively, are as follow:



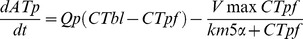
.
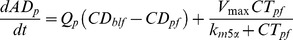



With the replacement of the Michaelis-Menten term with reaction rate kinetic terms, the equations in FM are now given by:
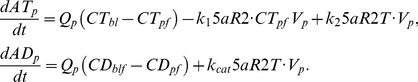
(3)


Descriptions for all state variable and parameter abbreviations in equations are found in Tables 1, 4, 5 and 6.

**Table 5:** State variables for equations (3) – T metabolism in the prostate.

Variable	Description	Units
*AT_p_*	Amount of T in prostate	nmol
*CT_pf_*	Free concentration of T in prostate	nM
*AD_p_*	Amount of DHT in prostate	nmol
*CD_pf_*	Free concentration of DHT in prostate	nM

**Table 6:** Parameters for equations (3) – T metabolism in the prostate.

Parameter	Description	Units	Value
*Q_p_*	Blood flow rate to prostate^1^	L/hr	variable
*V_p_*	Total mass of prostate^2^	kg	variable

1 Blood flow rate to prostate is a function of prostate mass; see [36] for details.

2 Total mass of prostate is the total of basal and androgen sensitive cellular and ductal lumen masses [36]. In PM, prostate volume and mass were used interchangeably, assuming 1 g of ventral prostate tissue equals 1 mL. In this manuscript, we consistently refer to prostate measurement in mass, although we continue to use the original symbol *V_p_* used in PM.

#### Revisiting total DNA binding sites allotted in PM

In PM, we assumed an equal number of DNA binding sites for each of the four genes: anti-apoptosis, fluid production, cell proliferation and 5aR2 production. Upon revisiting this assumption and noting that some processes such as fluid production, apoptosis protection and cell proliferation obviously involve far more than a single gene (as is the case for 5aR2 production), and hence many more DNA sites relative to 5aR2 production, we decreased the number of total DNA binding sites for 5aR2 by 75%. The other three genes still contain the same number of DNA sites, and these numbers have not changed from PM. In the process of making this change in FM, it was discovered that the number of DNA binding sites can be significantly changed (an order of magnitude or more) without considerable changes in model behavior and with no parameter adjustments. This is due to the fact that the concentrations of available dimers in the prostate are much greater (approximately 40 fold) than the total number of DNA binding sites. Therefore, gene occupancy is mainly driven by binding rate constants. This feature allows one to reasonably adjust the number of DNA binding sites for each of the four genes to fit a variety of assumptions. For simplicity, we limited the assumptions in FM to those just noted.

#### Alteration to equation for cellular mass of prostate

In PM, we defined basal (androgen insensitive) and androgen sensitive portions of the prostate. This is further fractionated between cellular mass and ductal lumen mass. The original equation for the androgen sensitive cellular portion of the prostate mass (*VPC_1_*) in PM is given by:
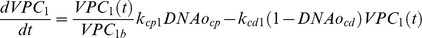
where *VPC_1b_* is the steady-state (initial) value of *VPC_1_*. However, preliminary analysis of FM with this equation revealed the term 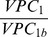 dominates the growth rate of the equation under noncastrated conditions. This hinders the ability of the prostate to regrow under finasteride-treated conditions, which is critical for treatment simulations. Under castrated conditions, the term *DNAo_cp_* dominates, and so the term 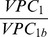 has no significant influence, and hence PM, a model of castration, is unaffected whether the term is present or absent in the equation. From the standpoint of physiology, it is known that factors influencing tissue growth are complex. With or without the term 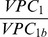 in the equation, the growth rate represented in the equation is a simplification of the physiological process. Therefore, in FM the term was dropped and the new equation is given as:



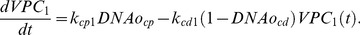
(4)Descriptions for all parameter abbreviations are found in Table 7.

**Table 7:** Parameters for equation (4) – prostate mass.

Parameter	Description	Units	Value
*k_cp1_*	Prostate cell proliferation rate constant^1^	mg/hr	0.5
*k_cd1_*	Prostate cell apoptosis rate constant^2^	hr^−1^	-
*VPC_1b_*	Steady state androgen sensitive prostate cellular mass^3^	mg	191
*DNAo_cp_*	Prostatic cell proliferation gene occupancy^4^	fraction	variable
*DNAo_cd_*	Prostatic anti-apoptotic gene occupancy^4^	fraction	variable
*VPC_2_*	Basal prostate cellular mass^1,5^	mg	18
*VPL_2_*	Basal prostate ductal lumen mass^1,5^	mg	18.2
*k_DNAoffTT_*	Relative DNA potency for TT:AR dimers^1^	-	1.6

1 New value for FM; see text.

2 Forced steady state rate constant; see Text S2.

3 New steady state value for FM.

4 Algebraic variable; see Text S1.

5 In [36], prostate volume and mass were used interchangeably, assuming 1 g of ventral prostate tissue equals 1 mL. In this manuscript, we consistently refer to prostate measurement in mass, although we continue to use the original symbols *VPC_2_* and *VPL_2_* used in PM.

#### Finasteride tissue exposure estimation

**Figure 3:**
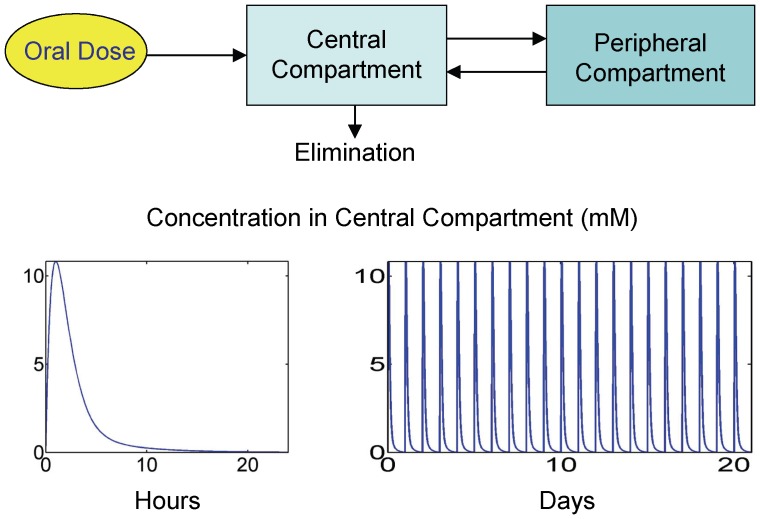
Finasteride pharmacokinetic (PK) model as reported in Stuart, *et al.* (2001) [26]. A 2-compartment model was described (top figure) with linear absorption and elimination terms. The equation for the central compartment was modified in FM to account for finasteride binding to prostatic 5aR2 (see the differential equation list for FM in Text S3). The bottom-left plot depicts a single, 40 mg/kg dose, PK profile over 24 hours. The bottom-right plot depicts daily 40 mg/kg dosing for 21 days, corresponding to the experimental conditions described in [42]. Plots depict total plasma finasteride concentrations.

A compartmental PK model for finasteride was developed for rats by Stuart and coworkers using a two-compartment model with linear rate constants to describe PK time profiles [26]. By the nature of small molecule characteristics, it was assumed in FM that free plasma concentrations of finasteride are approximately in equilibrium with free concentrations in well-perfused tissues, such as the prostate and liver. Therefore, the compartmental PK model from Stuart [26] was used to approximate prostate and liver exposure of finasteride, using free concentrations predicted in the central compartment of the model, *F*. Finasteride has been shown to be approximately 90% bound to proteins in human plasma [41]. Since finasteride plasma protein binding in rat was not found to be reported in the literature, it was assumed in this model that the percent of rat and human protein binding is the same. In the FM model, only unbound finasteride in the central compartment is available for binding to 5aR. The finasteride PK model is depicted in Figure 3. Equations for this model are available in Text S3, Table S5.

### Calibration of FM

**Figure 4:**
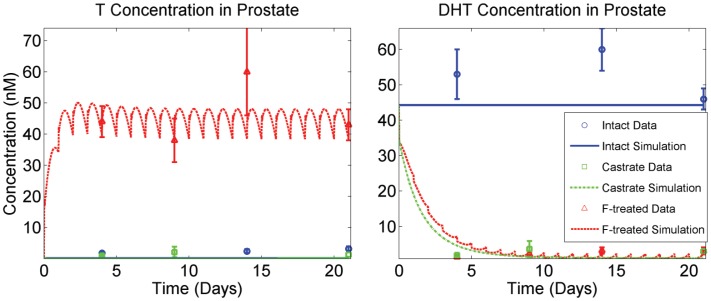
Model fits to prostatic T and DHT concentration data described in the Rittmaster data set. Plots depict model fits to free androgen concentration in prostate under three experimental conditions: intact, castrated and finasteride (F)-treated (daily dosing for 21 days at 40 mg/kg). Under the experimental conditions described in [42], nearly complete depletion of DHT is observed (data and model) in the prostate (right panel), while prostatic T concentrations are significantly elevated in a compensatory capacity due to the LH-T/DHT feedback loop (left panel).

**Figure 5:**
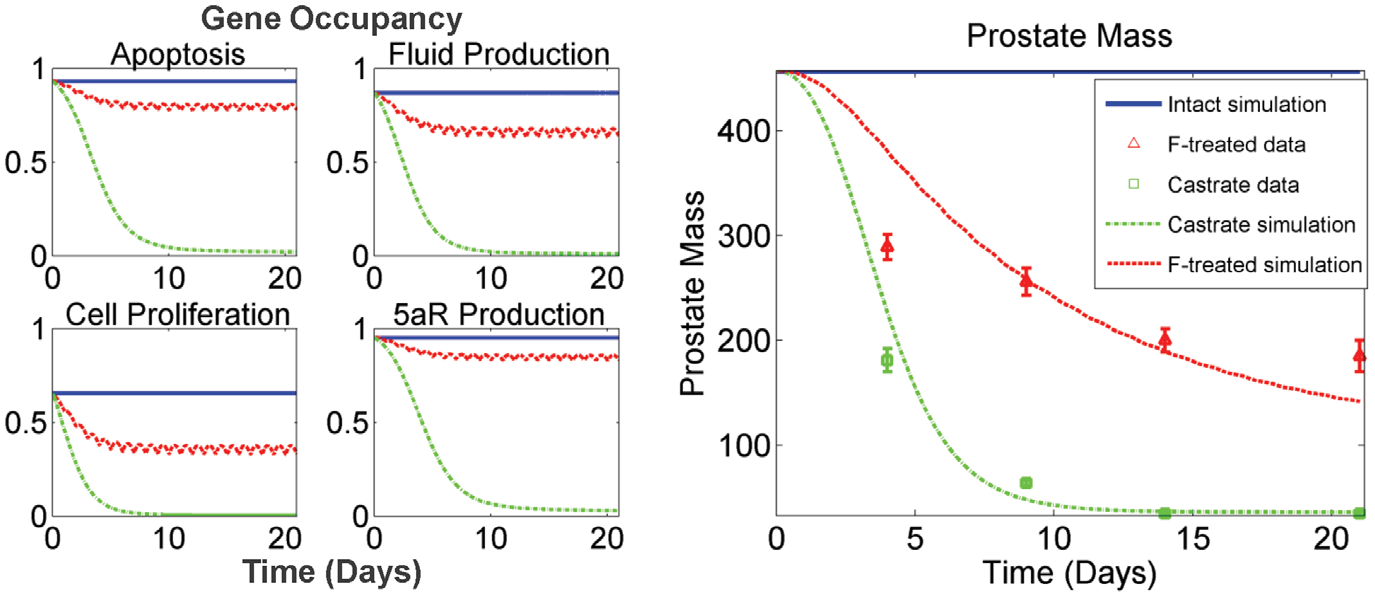
Downstream effects of prostatic hormone kinetics. (Left panel) Model simulations of time courses for gene occupancies (as fractions of complete occupancy) for anti-apoptosis, fluid production, cell proliferation, and enzyme (5aR2) production. Note the substantially larger effect castration is predicted to have on the anti-apoptosis and enzyme production genes. (Right panel) Model fits to prostate mass data (mg) described in [42] under three experimental conditions: intact, castrated and finasteride (F)-treated (daily dosing for 21 days at 40 mg/kg). Intact prostate mass is 457 mg (average, based on Rittmaster data), depicted at the very top of the plot. When DHT is nearly depleted from F dosing (see Figure 4), elevated T levels are not capable of maintaining intact prostate mass (right panel).

For equations that were changed and/or added to accommodate the expanded capabilities of FM, several approaches were taken to obtain the new parameters. First, experimentally obtained values available in the literature were used when possible. Second, we analyzed the model to determine which of the remaining unknown parameters its predictions are sensitive to, and then used a set of data for finasteride-treated rats available from Rittmaster and coworkers to identify the parameter values through modeling fitting [42]. This data set includes prostatic T and DHT concentrations (Figure 4) as well as prostate masses for intact, finasteride-treated and castrated rats (Figure 5). The rats were treated with 40 mg/kg daily for 21 days and sampling time points were taken at 4, 9, 14 and 21 days. The insensitive parameters were set to physiologically plausible values. PM was originally fit to multiple data sets for prostate regression due to castration. During the fitting process for FM, we found that several existing parameters in the unchanged equations needed to be adjusted, in order to specifically fit the Rittmaster data set.

The model was calibrated to produce biologically plausible behavior with respect to pharmacokinetics, steady-state behavior, and the dynamics of the prostate under the various study conditions with the goal of maintaining good model agreement with the Rittmaster data set while changing as few existing parameters from PM as possible. Thus, for example, the degradation rate for the free androgen receptor in PM results in an improbably short half life compared with one literature value of 3 hours in cells [43], but we chose to keep the existing PM value because recalibration of other parameters would be needed to retain consistency with the data originally evaluated. All model state variables and parameters that are new for FM are listed in Tables 1, 2, 3, 4, 5, 6, 7 with values and references (where applicable). All other equations, state variables and parameter values are identical to those reported previously [36] and are available in Tables S1, S2, S3, S4.

#### Adjusting the existing parameters

Model calibration began with the castrate data in the Rittmaster data set, since castration results in nearly immediate depletion of androgens in the system, and therefore none of the unknown parameters in FM are either sensitive or informed by this experimental condition. PM was fit to multiple data sets of prostate regression for castrated rats. It was observed that after complete regression, a small fraction of the prostate remained in the absence of androgens. This fraction was called the basal mass, and was separated into two parts – basal cellular mass and basal ductal lumen mass. The Rittmaster data set shows a significantly smaller basal prostate mass after castration than the average of the multiple data sets to which PM was fit [42]. Hence, the parameters governing the size of the basal cellular and ductal lumen masses in FM, *VPC_2_* and *VPL_2_* respectively, were adjusted so that FM fits the castration-driven regressed prostate observed in the Rittmaster data set (see Figure 5, second panel). The parameter *k_cp1_* (prostate cell proliferation rate constant) was also adjusted to the Rittmaster castrate data in order to adequately capture the rate of regression of the prostate after castration. Once FM was in agreement with the Rittmaster castrate data, we fit the unknown parameters to the finasteride-treated data. In the fitting process, it was found that one additional existing parameter, *k_DNAoffTT_* (relative potency for TT dimers bound to DNA sites – see [36]), is critical for model agreement with the finasteride-treated data set. The original value for this parameter in PM was 6.0. However, at this value, TT dimers are not effective enough to maintain the necessary gene occupancies that correlate with the rate of prostate regression observed in the Rittmaster data set. The result was a significant over-determination of prostate regression due to finasteride treatment. Therefore, in FM this value was adjusted to 1.6, which allowed the unknown parameters to be appropriately fitted to the finasteride-treated data (see below and Figure 5).

#### Fitting the unknown parameters

Although values for the majority of the new parameters for FM were available in the literature, some values were not found and remained unknown. A sensitivity analysis was conducted to determine the sensitivity of FM to all new parameters to guide our efforts in estimating unknown values. The algorithm used for the analysis is the well-established method of control coefficients successfully utilized by Lee and coworkers in the mathematical modeling of the Wnt pathway [44]. Control coefficients were calculated in the following manner:
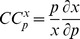
and the computational approximation (*CCA*) to this equation was formed using the finite difference method, yielding:



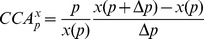
where *x* is a state variable of FM calculated at the end of the 21-day simulation, *p* is a parameter of FM. The element Δ*p* is a 1% increase in the value of *p*, which has been shown to be the most numerically stable quantity of variation for this type of sensitivity calculation. Details on analysis and interpretation of control coefficients can be found in [44]. Briefly, the larger in magnitude the value the control coefficient takes, the more sensitive the state variable is to the parameter. A positive control coefficient indicates the parameter has a positive effect on the state variable. That is, an increase in the parameter value results in an increase in the value of the state variable. Conversely, a negative control coefficient indicates the parameter has a negative effect on the state variable. Table 8 depicts the results of the analysis. In an effort to keep the analysis manageable, only state variables directly associated with the additions made to create FM were analyzed for sensitivity, and only those parameters that are new to FM or whose values have been changed from the original model upon development of FM were included in the results. Furthermore, only control coefficients with values greater than 0.2 are reported in the table, since state variables with control coefficients below this level are considerably insensitive.

**Table 8:** Sensitivity analysis for FM parameters.

Parameter	Sensitive State/Algebraic Variable				Control Coefficient (*CCA*)					Parameter Source
*K_m_*	*CD_p_*				−0.72					[26]
*k_dg_*	*CD_p_*				0.81					Fit^2^
*K_m5a_*	*CT_bl_*	*CD_bl_*	*CT_p_*	*V_p_*	0.26	−0.3	0.21	0.22		[26]
*k_5_*	*CD_p_*				−0.72					[26]
*k_cat_*	*CD_p_*				0.82					[39]
*K_i_^5aR2^*	*CD_p_*				0.72					[26]
*K_i_^5aR1^*	*CD_bl_*				0.21					[26]
*k_DNAoffTT_*	*DNAo_cd_*	*DNAo_sec_* 1	*DNAo_cp_*	*V_p_*	−0.22	−0.36	−0.65		−1.1	Fit^3^

1 Algebraic variable for prostatic fluid production gene occupancy.

2 Parameter was directly fit by calibrating model to prostatic DHT data (see Figure 4).

3 Parameter was directly fit by calibrating model to prostate volume data (see Figure 5).

The only parameters we tested that are not reported in Table 8 are *k_1_*, *k_3_* and *k_6_*. All analyzed state variables in FM were found to be insensitive to these parameters. Physiologically plausible values were chosen for *k_1_* and *k_3_* (see Table 4), and the value for *k_6_* is reported in [26]. It is worthy to note that, although FM is not sensitive to *k_1_* and *k_3_*, it is sensitive to *K_m_* and *K_i_^5aR2^*. This suggests that although the system is insensitive to *k_on_* and *k_off_* values for 5aR2 binding to T and F, the ratio is important. The only parameters in Table 8 whose values are not directly taken from literature sources are *k_dg_* and *k_DNAoffTT_*. However, these parameters were directly and uniquely fitted to specific data in the Rittmaster data set (and the calibration of *k_DNAoffTT_* is discussed above). When fitting *k_dg_* to prostatic DHT concentrations (see Figures 4 and 5), we found that the value was dependent on the value of *K_m_* (data not shown). That is, identical fits to the data can be obtained for varying values of *K_m_* by adjusting the value of *k_dg_*. Since the *K_m_* values for both 5aR1 and 5aR2 and most of the other kinetic parameters for 5aR2 used in the model were determined in the Stuart laboratory, these values were used in attempt to ensure comparability. Using the value of 0.6 nM for *K_m_* in FM, the fitted value of *k_dg_* was found to be 0.016 hr^−1^, yielding a biologically plausible half life of 5aR2 of approximately 2.6 days. Figures 4 and 5 depict model fits to the Rittmaster data set using the above parameter values. In Figure 5, the left panel shows gene occupancy predictions corresponding to the three experimental conditions in the Rittmaster data set – intact, castrated and finasteride treated. These gene occupancies drive the prostate mass dynamics observed in the right panel of Figure 5. With these values, FM is still in complete agreement with the kinetics and dynamics represented in Figure 10 of [36] (see Figure S1), which shows the kinetics of blood T concentration, prostatic androgen receptor (AR) concentration, and prostate mass dynamics following castration. As described above, prostate mass dynamics in FM are calibrated specifically for the Rittmaster data set, which results in a slight difference in fit to the multiple data sets used in calibrating PM.

Finally, the Rittmaster lab reported TUNEL staining data quantifying apoptosis in prostatic epithelial cells of intact, castrated, and 4-day, daily finasteride treated rats by observing the percent of cells that stained positively for apoptosis [45]. While there is no direct way to relate these data to a specific model output, the trend in the data are consistent with the ordering observed in FM for anti-apoptotic gene occupancy (Fig. 5): castrate > finasteride-treated > intact. Furthermore, the data showed that castration causes a far greater effect on apoptosis than treating with finasteride, compared with intact data. This observation is once again in agreement with the kinetics of anti-apoptotic gene occupancy in FM (Figure 5).

#### FM steady-state predictions agree with experimental data

With the new equations and parameters set, we checked the predicted steady-state levels of prostatic androgens and 5aR2 against experimental data. Figure 4 shows model agreement with intact prostatic levels of T and DHT reported by the Rittmaster data set. To confirm the forcing of *E_syn_ = E_deg_* at steady state, we checked model predictions for free prostatic 5aR2 at steady-state (19 nM) and 5aR2 bound to T (1 nM). Recalling the calculated estimate for total prostatic 5aR2 of 20 nM and noting there are no finasteride-bound complexes at steady state, the forcing rule functions correctly.

### FM Predicts Unexpected Hormone Kinetics

#### Hormone kinetics and downstream effects predicted by FM

**Figure 6:**
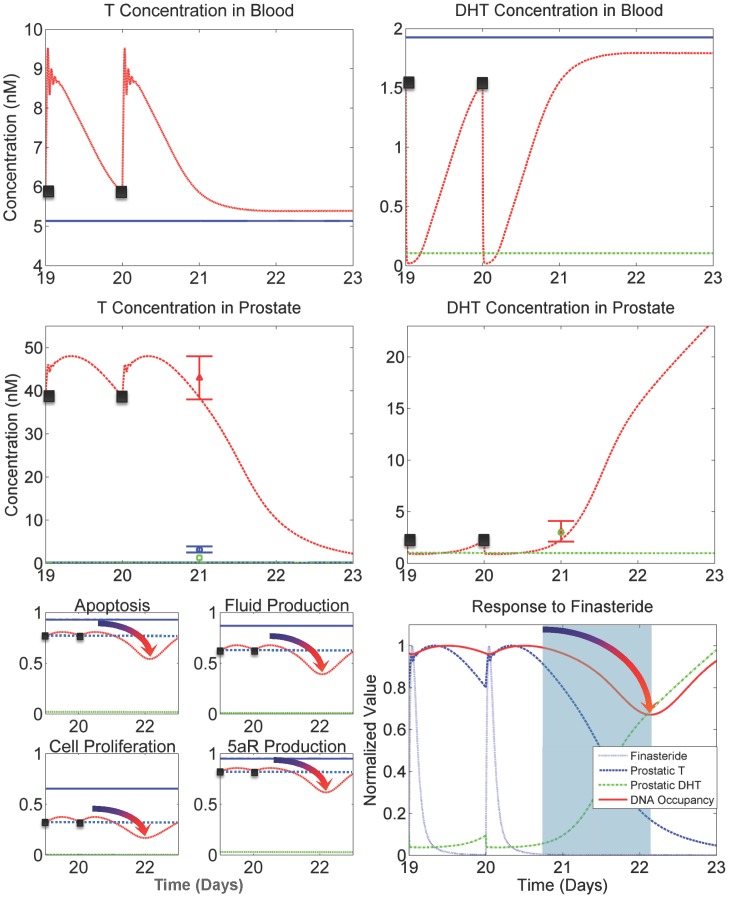
Kinetics of T and DHT and their predicted effects on prostatic gene occupancy. Black points in the plots represent the 20^th^ and 21^st^ (last) finasteride dose in the simulation. The plots extend three days in time after the last dose to capture rebound effects. (Top right panel) Blood DHT drops quickly in response to plasma finasteride exposure, mainly due to liver 5aR1 inhibition. (Top left panel) Blood T responds quickly to decreasing blood DHT concentrations via LH signaling in the testicular-pituitary axis. (Middle right panel) Prostatic DHT concentrations are decreased due to dropping levels of blood DHT and prostatic 5aR2 inhibition. (Middle-left panel) Prostatic T concentrations increase due to rising blood T levels and decreased 5aR2 activity. (Bottom left panel) Gene occupancies demonstrate the significant influence of T and DHT kinetics on downstream events. Note that gene occupancies increase between doses 20 and 21 (black dotted line depicts gene occupancy level at time of dose), yet after the increase from the last dose the occupancy significantly decreases before finally increasing to return to baseline (intact value). This behavior is shown in the plots by the gradient-shaded arrows. (Bottom-right panel) The kinetics of finasteride, prostatic T and DHT, and gene occupancy (anti-apoptosis depicted) are shown simultaneously in a single time course (values were normalized for scaling purposes). The kinetics of gene occupancy are directly tied to the combination of kinetics between T and DHT. The rise in occupancy between doses is caused by the rise in prostatic T. The light blue shaded region shows where T and DHT are collectively at their lowest point in the time course. This is followed by the observed decrease in gene occupancy, which takes place well after the drug has significantly cleared.

To investigate predicted hormone kinetics of finasteride exposure, we used FM to simulate 21 days of finasteride dosing (40 mg/kg/day), consistent with the experimental protocol from the Rittmaster data set, followed by a three-day washout period. This allows predictions of rebound effects after the drug has significantly cleared the system. Figure 3 and Figure 6 (bottom-right panel) show finasteride plasma kinetics, which drive 5aR inhibition in the liver and prostate. Figure 6 also depicts blood and prostatic androgen kinetics as well as gene occupancy time profiles for Days 19 through 23 under intact, castrated and finasteride treated conditions. The plots show the final two doses followed by predictions for a three-day washout, which was not included in the experimental protocol. In the top-right panel, blood DHT concentrations rapidly decrease in the finasteride treated group mainly due to the inhibition of 5aR1 in the liver. In the top-left panel, blood T concentrations rapidly increase due to both a buildup of T from 5aR inhibition (and hence less DHT being formed) and response from the feedback in the testicular-pituitary axis between LH and T/DHT. When T and/or DHT levels drop in blood, LH signals the testes to produce more T in response. At the peaks of the T concentration curve, decaying oscillations are seen, which are due to the LH-T/DHT feedback loop present in the physiologically-based pharmacokinetic model (PBPK), depicted in Figure 2. These oscillations demonstrate very quick and sensitive response to DHT modulation in the system, which is consistent with the behavior seen in PM (see Figure 5 in [36]). We have been unable to experimentally confirm this behavior, either in the laboratory or in the literature, due to the hindering proximity between time points that would be necessary to observe these kinetics in a study.

The right-center panel depicts prostatic DHT concentrations, where the finasteride treated group is responding to finasteride exposure. Concentrations for the intact group are not displayed due to axis bounds, but are shown in Figure 4. The left-center panel shows kinetics of prostatic T concentrations. In the finasteride treated group, the decaying oscillations seen in the blood T concentration profile are observed to slightly affect the prostatic T concentration curves. The effect is muted due to distribution kinetics of T from blood to the prostate in the PBPK model for T. From these kinetics, it is seen that prostatic androgen kinetics are predicted by FM to be driven mainly by 5aR2 activity in the prostate, and only mildly affected by 5aR1 activity in the liver. FM also predicts that prostatic T response to decreased DHT is very rapid and pronounced (see Figure 4). Furthermore, the magnitude of T response from minimum to maximum (see Figure 6 center-left panel), where minimum occurs at each dose (x = 19, 20) and maximum occurs approximately 7.5 hours after, is considerable relative to DHT response (Figure 6, center-right panel). A predicted *increase* in gene occupancy between doses of finasteride (see Figure 6, bottom-left panel) is an unexpected result, which suggests the balance between prostatic T and DHT kinetics is critical in optimal finasteride dosing regimens. Investigating further, we see in the lower-right panel of Figure 6 that the kinetics of T and DHT due to finasteride exposure work in concert to drive the response of gene occupancy. The observed increase in gene occupancy after a finasteride dose (which is not intuitive) is due to the increase in T in response to decreasing DHT levels. It is only after T significantly decreases in response to increasing DHT levels that gene occupancy begins to significantly drop before returning back to baseline (depicted in the shaded region of the plot). The potential implications of this prediction are useful, in that the model is suggesting an alternative 40 mg/kg dosing schedule in rats could produce a greater decrease in prostate size by taking advantage of this delayed effect on gene occupancy.

#### FM suggests an optimal relationship between dosing and frequency

**Figure 7:**
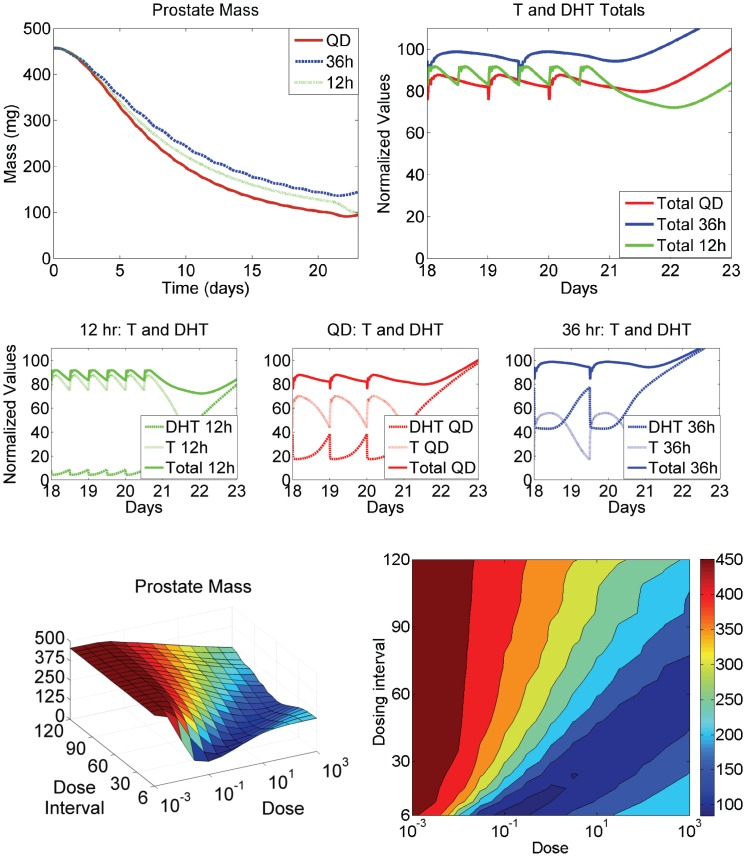
Analysis of hormone kinetics in relation to prostate volume, finasteride dosing amount and frequency. FM suggests alternative dosing schedules may increase the effect of finasteride on prostate volume. (Top left panel) a 40 mg/kg dose of finasteride is predicted to result in different amounts of prostate regression, depending on dosing frequency. Against intuition, a QD (daily) dose results in greater regression than a dose given every 12 hours. A dose given every 36 hours results in nearly as much regression as QD dosing. (Middle panels) For 12-hr, QD and 36-hr dosing, prostatic T and DHT levels were normalized against their affinities for AR (see main text) and were plotted over the last 4 days of dosing. The plots demonstrate the differing effect each dosing frequency has on T and DHT. The solid lines represent the sum of normalized T and DHT to comparatively depict the signal strength for prostate maintenance. (Top right panel) The normalized sums of prostatic T and DHT for each dosing frequency are depicted on the same plot, showing why the QD dosing schedule is the most effective in prostate regression. On average, the normalized sum amounts to the least signal of the three schedules, resulting in the most significant decrease in prostate volume. (Bottom right panel) FM was run systematically, varying both dose and dose frequency. The plot depicts the relationshop between dose, dose frequency and prostate volume at the end of 21 days of dosing. The suggestion of an optimal relationship between dose and dose frequency, depicted by the trough (deep blue color), is seen. (Bottom right panel) The 3-D surface was projected onto a 2-D plane for easier visualization of the optimum dose/frequency relationship.

In light of the predicted prostatic androgen kinetics shown in Figure 6, the question could be asked if alternative dosing and/or dose scheduling could be found by FM, where the kinetics of prostatic T and DHT are more optimally balanced to maximize loss of gene occupancy by androgen:AR dimers. We found through simulation that FM indeed suggests differing degrees of rat prostate regression by altering the dosing schedule (Figure 7, top-left panel). Surprisingly, FM suggests QD (24-hour) dosing outperforms 12-hour dosing, and 36-hour dosing is almost as effective as the other two dosing schedules.

Closer inspection of prostatic T and DHT kinetics hold the key to understanding the prediction. Figure 7, middle 3 panels, depict prostatic T and DHT concentrations over the last 4 days of dosing of a 21-day dosing period at 40 mg/kg. Each plot represents each dosing schedule considered. The concentrations for T and DHT shown are normalized by their respective dissociation constants for AR, to comparatively depict their relative contributions to signaling gene transcription. The solid lines in the plots represent the normalized sum of T and DHT concentrations to depict the total relative signal for gene transcription. Figure 7, top right panel depicts the normalized sums for each dosing schedule. From this plot, it is clear to see why in this case QD dosing outperformed the other schedules. On average, its normalized total signal is lowest among the three schedules, resulting in an increase in prostate regression over the other schedules. Although the very end of dosing results in a significant drop in total signal for the 12-hour dose group, which corresponds to a significant drop in prostate volume (Figure 7, top left panel), it is clear from the kinetics that prior to the last dose, the 12-hour dosing schedule keeps enough pressure on prostatic DHT, forcing a significant increase in T concentration, that total signal remains higher in the 12-hour group than QD dosing.

From this analysis, we investigated the concept of an “optimal” relationship between dose and dosing interval for rat prostate regression. In order to see this relationship, we systematically ran FM simulations with varying doses and dosing schedules to map a three-dimensional surface for prostate volume as a function of dose and frequency. Figure 7, bottom left panel depicts the resulting surface, where a clear trend (dark blue) is seen. Figure 7, bottom right panel is a projection of the 3-D surface onto a 2-D plane, for easier visualization of the optimal dose/frequency relationship.

## Discussion

Changes in prostate size are of concern from both a clinical perspective and from the perspective of toxicological studies, which are typically conducted using rats. Predicting how drugs or environmental chemicals impact the underlying hormonally-mediated regulation of prostate size and function is intrinsically a multiscale problem, which requries various modeling methodologies. Processes range from the molecular level, e.g., androgen receptor binding ligand and DNA response elements or 5aR inhibitor binding its target, to the cellular level, e.g., effects of altered DNA regulation on cell proliferation, apoptosis, and prostatic fluid production, to the tissue level at which resulting changes in prostate mass occur, to the organ system level at which the testicular-pituitary axis provides central hormonal feedback regulation. The considerably different scales associated with these processes result in a combination of molecular kinetic modeling (see Figure 1), tissue-level dynamic modeling and several PBPK model structures (see Figure 2 and [36]). The model presented here expands previous modeling efforts for the central axis and prostatic regression following castration [36], [46] to include changes with 5aR inhibitor dosing. Inhibition of 5aR results in decreases in prostatic and blood DHT but increases in prostatic and blood T, which can only be adequately described in this multiscale context. Furthermore, this extension demonstrates the utility of this biological system platform to incorporate activities of greater breadth and/or depth addressing additional aspects of pharmacology and biology.

The utility of this expanded model in preclinical and, if scaled to human, clinical settings is that investigators can gain quantitative understandings and test and generate various hypotheses of different mechanisms of inhibition (i.e., 5aR competitive and time-dependent inhibition) and alternative dosing regimens (see Figures 6 and 7). The alternative dosing regimens proposed by the rat model could either be evaluated by creating a human version of the model or, if one were developing a new compound, might be explored directly in preclinical studies and clinical trials. It also provides insights into the relative importance of T metabolism in the liver vs. prostate. Under control conditions, blood levels of DHT in the adult male rat are very low in comparison with blood T levels or prostatic DHT levels, suggesting T metabolism in the prostate is the main driver of intraprostatic DHT levels. With the quantitative aid of FM, it might be determined that inhibiting 5aR in the liver is negligible with respect to effects on prostate size. While the simulations discussed are for rats and a compound already in therapeutic use, thes types of hypotheses would influence every phase of a 5aR inhibitor project from discovery through clinical development.

As complex as the current model is, it remains a substantial simplification of the true biology and the ability of drugs or environmental chemicals to perturb prostatic function. The model of Eikenberry and coworkers focuses on the potential to describe the evolution of cells under varied androgen exposures that might result in transitions to cancer and altered regulation of androgen receptor levels impacting the success or failure of antiandrogen oncology treatments [34]. In both PM and FM, androgen-dependent regulation of receptor levels is not yet considered. However, this may be an ideal example of another useful expansion of FM, since the mechanisms associated with the regulation and dysregulation of prostatic AR have been implicated in androgen-independent prostate cancer for over a decade [33], [47], [48], [49], [50], [51]. There is currently no effective standard of care for this form of cancer, and it is associated with an exceptionally poor prognosis. Perhaps critical mechansistic insights may be gained in quantitatively understanding the roles of hormones and key proteins that lead to ligand-independent activation of AR, such as heat shock proteins, cofactors and signals downstream of important cell surface receptors like receptor tyrosine kinases. An effort toward this approach has recently been published by Jain and coworkers [52], where they developed a model for prostate cancer and its progression and included “personalized” parameters to account for patient variability and the inherent heterogeneous nature of cancer tumors. This work highlights the question of benefits vs. drawbacks of intermittent dose schedules for various therapeutics. Although this work nicely captures the cancer-specific relationships between androgens, AR, and prostate-specific antigen (PSA), and widely used and accepted diagnostic marker for prostate cancer and progression, it still does not quantitatively capture the multi-scale, multi-tissue nature of the feedback and compensatory relationship between T and DHT. Ideally, one could potentially combine a correctly scaled form of FM and the Jain model for a comprehensive look at the role of 5aR in prostate cancer. Particularly, if the feasibility of creating a human parameterization is realistic, one might conceive a role for FM in a potential combined role with the Jain model in a translational context, where more accurate predictions of human response to therapeutic intervention could be made, based on *in vitro* and/or *in vivo* preclinical data.

The simplifications utilized in the current model can be informative. Cell proliferation, protection from apoptosis, and fluid production are multigene processes, but simple empirical relationships between a single androgen-regulated gene and each of these activities have been described for conditions of prostate regression following castration and androgen reductions via 5aR inhibition. Available data in the literature on prostate maintenance or regrowth with androgen supplementation following castration represent another area for expansion of the model. An initial evaluation of prostate regrowth indicated that the cell proliferation rate would need to be increased to fully capture the growth dynamics (results not shown). This suggests additional biological mechanisms should be incorporated for (at least) the regulation of cell proliferation.

This multiscale model provides a useful framework for incremental and modular expansions of its description of androgen-dependent biology occurring in multiple tissues. It integrates existing published scientific literature permitting evaluation of their consistency and it provides predictions that can be experimentally tested, such as those here for alternative dosing strategies. Ultimately, FM and any subsequent augmentations could serve as supplemental tools for drug target validation by facilitating quantitiative, causal linkages between target, mechanism and outcome. Furthermore, these models can play a central role in identifying the most informative biomarkers in the system to measure and in the translational strategy for predicting human response from preclinical data.

## Materials and Methods

The model was implemented in MATLAB 2009b (The Mathworks, Natick, MA). The package algorithm *ode15s* was used to solve the system of ordinary differential equations, with relative tolerance set to 10^−4^ and absolute tolerances set to 10^−6^. Sensitivity analysis was conducted as specified in [44], based on the concept of control coefficients, which were originally proposed for use in metabolic networks by Heinrich and Schuster [53] as well as Fell and coworkers [54]. To create the 3-D plot depicted in Figure 7, bottom left panel, doses were chosen by starting at 0.001 mg/kg and making half-log increments up to 1000 mg/kg. Dosing intervales were chosen by starting at dosing every 6 hours, and increasing the interval by 6 hours up to 120 hours. Then FM was run in every combination of the above doses and intervals and prostate volumes were recorded at the end of 21-day dosing periods. The prostate volume data were then entered into a grid as functions of dose and interval.

## Supporting Information

Figure S1 FM captures kinetics dynamics of system following castration.
(DOC)

Table S1 State variables for FM.
(DOC)

Table S2 Model parameters – physiological.
(DOC)

Table S3 Model parameters – Hormone pharmacokinetics and non-prostatic metabolism.
(DOC)

Table S4 Model parameters – Prostate biochemistry and metabolism.
(DOC)

Table S5 Model parameters – Finasteride pharmacokinetic (PK) model.
(DOC)

Text S1 Calculation of the algebraic variables for gene occupancy in FM.
(DOC)

Text S2 Forced steady state on prostate cell growth and death dynamics.
(DOC)

Text S3 Differential Equations for FM.
(DOC)

## References

[1] Kholodenko B, Yaffe MB, Kolch W (2012) Computational approaches for analyzing information flow in biological networks. Sci Signal 5: re1. 22510471 10.1126/scisignal.2002961

[2] Hunter PJ, Borg TK (2003) Integration from proteins to organs: the Physiome Project. Nat Rev Mol Cell Biol 4: 237–243. 12612642 10.1038/nrm1054

[3] Westerhoff HV, Palsson BO (2004) The evolution of molecular biology into systems biology. Nat Biotechnol 22: 1249–1252. 15470464 10.1038/nbt1020

[4] Clark LH, Schlosser PM, Selgrade JF (2003) Multiple stable periodic solutions in a model for hormonal control of the menstrual cycle. Bull Math Biol 65: 157–173. 12597121 10.1006/bulm.2002.0326

[5] Selgrade JF, Harris LA, Pasteur RD (2009) A model for hormonal control of the menstrual cycle: structural consistency but sensitivity with regard to data. J Theor Biol 260: 572–580. 19560471 10.1016/j.jtbi.2009.06.017

[6] Pozzi P, Bendotti C, Simeoni S, Piccioni F, Guerini V, et al (2003) Androgen 5-alpha-reductase type 2 is highly expressed and active in rat spinal cord motor neurones. J Neuroendocrinol 15: 882–887. 12899683 10.1046/j.1365-2826.2003.01074.x

[7] Torres JM, Ortega E (2003) Precise quantitation of 5alpha-reductase type 1 mRNA by RT-PCR in rat liver and its positive regulation by testosterone and dihydrotestosterone. Biochem Biophys Res Commun 308: 469–473. 12914773 10.1016/s0006-291x(03)01423-2

[8] Orlowski J, Bird CE, Clark AF (1988) Androgen metabolism and regulation of rat ventral prostate growth and acid phosphatase during sexual maturation. J Endocrinol 116: 81–90. 3339295 10.1677/joe.0.1160081

[9] Grino PB, Griffin JE, Wilson JD (1990) Testosterone at high concentrations interacts with the human androgen receptor similarly to dihydrotestosterone. Endocrinology 126: 1165–1172. 2298157 10.1210/endo-126-2-1165

[10] Gubbay J, Doyle JP, Skinner M, Heintz N (1998) Changing patterns of gene expression identify multiple steps during regression of rat prostate in vivo. Endocrinology 139: 2935–2943. 9607804 10.1210/endo.139.6.6075

[11] Isaacs JT (1984) Antagonistic effect of androgen on prostatic cell death. Prostate 5: 545–557. 6483690 10.1002/pros.2990050510

[12] Nnane IP, Kato K, Liu Y, Lu Q, Wang X, et al (1998) Effects of some novel inhibitors of C17,20-lyase and 5alpha-reductase in vitro and in vivo and their potential role in the treatment of prostate cancer. Cancer Res 58: 3826–3832. 9731491

[13] Prins GS (1989) Differential regulation of androgen receptors in the separate rat prostate lobes: androgen independent expression in the lateral lobe. J Steroid Biochem 33: 319–326. 2779222 10.1016/0022-4731(89)90319-1

[14] Suzuki K, Ito K, Kurokawa K, Suzuki T, Shimizu N, et al (1997) Expression and degradation of rat androgen receptor following castration, testosterone replacement and antiandrogens administration: analysis by Western blot and immunohistochemistry. Tohoku J Exp Med 183: 159–172. 9550125 10.1620/tjem.183.159

[15] George FW, Russell DW, Wilson JD (1991) Feed-forward control of prostate growth: dihydrotestosterone induces expression of its own biosynthetic enzyme, steroid 5 alpha-reductase. Proc Natl Acad Sci U S A 88: 8044–8047. 1654556 10.1073/pnas.88.18.8044 PMC52442

[16] Berman DM, Russell DW (1993) Cell-type-specific expression of rat steroid 5 alpha-reductase isozymes. Proc Natl Acad Sci U S A 90: 9359–9363. 8415707 10.1073/pnas.90.20.9359 PMC47567

[17] Frye SV, Haffner CD, Maloney PR, Hiner RN, Dorsey GF, et al (1995) Structure-activity relationships for inhibition of type 1 and 2 human 5 alpha-reductase and human adrenal 3 beta-hydroxy-delta 5-steroid dehydrogenase/3-keto-delta 5-steroid isomerase by 6-azaandrost-4-en-3-ones: optimization of the C17 substituent. J Med Chem 38: 2621–2627. 7629802 10.1021/jm00014a015

[18] Habib FK, Ross M, Bayne CW, Grigor K, Buck AC, et al (1998) The localisation and expression of 5 alpha-reductase types I and II mRNAs in human hyperplastic prostate and in prostate primary cultures. J Endocrinol 156: 509–517. 9582508 10.1677/joe.0.1560509

[19] Normington K, Russell DW (1992) Tissue distribution and kinetic characteristics of rat steroid 5 alpha-reductase isozymes. Evidence for distinct physiological functions. J Biol Chem 267: 19548–19554. 1527072

[20] Thomas LN, Douglas RC, Lazier CB, Gupta R, Norman RW, et al (2008) Levels of 5alpha-reductase type 1 and type 2 are increased in localized high grade compared to low grade prostate cancer. J Urol 179: 147–151. 17997435 10.1016/j.juro.2007.08.155

[21] Tindall DJ, Rittmaster RS (2008) The rationale for inhibiting 5alpha-reductase isoenzymes in the prevention and treatment of prostate cancer. J Urol 179: 1235–1242. 18280514 10.1016/j.juro.2007.11.033 PMC2667246

[22] Frye SV, Bramson HN, Hermann DJ, Lee FW, Sinhababu AK, et al (1998) Discovery and development of GG745, a potent inhibitor of both isozymes of 5 alpha-reductase. Pharm Biotechnol 11: 393–422. 9760689 10.1007/0-306-47384-4_17

[23] Vermeulen A, Giagulli VA, De Schepper P, Buntinx A (1991) Hormonal effects of a 5 alpha-reductase inhibitor (finasteride) on hormonal levels in normal men and in patients with benign prostatic hyperplasia. Eur Urol 20 Suppl 1: 82–86. 1722168 10.1159/000471752

[24] Azzolina B, Ellsworth K, Andersson S, Geissler W, Bull HG, et al (1997) Inhibition of rat alpha-reductases by finasteride: evidence for isozyme differences in the mechanism of inhibition. J Steroid Biochem Mol Biol 61: 55–64. 9328210 10.1016/s0960-0760(97)00002-2

[25] Gisleskog PO, Hermann D, Hammarlund-Udenaes M, Karlsson MO (1999) The pharmacokinetic modelling of GI198745 (dutasteride), a compound with parallel linear and nonlinear elimination. Br J Clin Pharmacol 47: 53–58. 10073740 10.1046/j.1365-2125.1999.00843.x PMC2014202

[26] Stuart JD, Lee FW, Simpson Noel D, Kadwell SH, Overton LK, et al (2001) Pharmacokinetic parameters and mechanisms of inhibition of rat type 1 and 2 steroid 5alpha-reductases: determinants for different in vivo activities of GI198745 and finasteride in the rat. Biochem Pharmacol 62: 933–942. 11543729 10.1016/s0006-2952(01)00728-6

[27] Cohen YC, Liu KS, Heyden NL, Carides AD, Anderson KM, et al (2007) Detection bias due to the effect of finasteride on prostate volume: a modeling approach for analysis of the Prostate Cancer Prevention Trial. J Natl Cancer Inst 99: 1366–1374. 17848668 10.1093/jnci/djm130

[28] Kaplan SA, Roehrborn CG, Meehan AG, Liu KS, Carides AD, et al (2009) PCPT: Evidence that finasteride reduces risk of most frequently detected intermediate- and high-grade (Gleason score 6 and 7) cancer. Urology 73: 935–939. 19328538 10.1016/j.urology.2008.09.079

[29] Pinsky P, Parnes H, Ford L (2008) Estimating rates of true high-grade disease in the prostate cancer prevention trial. Cancer Prev Res (Phila) 1: 182–186. 19138954 10.1158/1940-6207.CAPR-07-0007

[30] Thompson IM, Goodman PJ, Tangen CM, Lucia MS, Miller GJ, et al (2003) The influence of finasteride on the development of prostate cancer. N Engl J Med 349: 215–224. 12824459 10.1056/NEJMoa030660

[31] Thompson IM, Tangen CM, Parnes HL, Lippman SM, Coltman CA Jr (2008) Does the level of prostate cancer risk affect cancer prevention with finasteride? Urology 71: 854–857. 18455628 10.1016/j.urology.2008.01.025 PMC2692669

[32] Xu Y, Dalrymple SL, Becker RE, Denmeade SR, Isaacs JT (2006) Pharmacologic basis for the enhanced efficacy of dutasteride against prostatic cancers. Clin Cancer Res 12: 4072–4079. 16818707 10.1158/1078-0432.CCR-06-0184

[33] Scher HI, Sawyers CL (2005) Biology of progressive, castration-resistant prostate cancer: directed therapies targeting the androgen-receptor signaling axis. J Clin Oncol 23: 8253–8261. 16278481 10.1200/JCO.2005.03.4777

[34] Eikenberry SE, Nagy JD, Kuang Y (2010) The evolutionary impact of androgen levels on prostate cancer in a multi-scale mathematical model. Biol Direct 5: 24. 20406442 10.1186/1745-6150-5-24 PMC2885348

[35] Suzuki R, Satoh H, Ohtani H, Hori S, Sawada Y (2010) Saturable binding of finasteride to steroid 5alpha-reductase as determinant of nonlinear pharmacokinetics. Drug Metab Pharmacokinet 25: 208–213. 20460827 10.2133/dmpk.25.208

[36] Potter LK, Zager MG, Barton HA (2006) Mathematical model for the androgenic regulation of the prostate in intact and castrated adult male rats. Am J Physiol Endocrinol Metab 291: E952–964. 16757547 10.1152/ajpendo.00545.2005

[37] Tenniswood M, Abrahams P, Winterton V, Bird CE, Clark AF (1982) Binding of testosterone, 5 alpha-dihydrotestosterone and 5 alpha-androstane (3 alpha- and 3 beta-), 17 beta-diols to serum proteins in the rat. J Steroid Biochem 16: 617–620. 7098477 10.1016/0022-4731(82)90096-6

[38] Turner TT, Nguyen QT (2002) Response of the adult prostate to prepubertal and postpubertal obstruction of the vas deferens in the rat. Urology 60: 186–190. 12100960 10.1016/s0090-4295(02)01673-4

[39] Bull HG, Garcia-Calvo M, Andersson S, Baginsky WF, Chan HK (1996) Mechanism-Based Inhibition of Human Steroid 5R-Reductase by Finasteride: Enzyme-Catalyzed Formation of NADP-Dihydrofinasteride, a Potent Bisubstrate Analog Inhibitor. J Am Chem Soc 118: 2359–2365.

[40] Segal IH (1975) Enzyme kinetics: behavior and analysis of rapid equilibrium and steady-state enzyme systems. New York: Wiley-Interscience. 957 p.

[41] Sudduth SL, Koronkowski MJ (1993) Finasteride: the first 5 alpha-reductase inhibitor. Pharmacotherapy 13: 309–325; discussion 325–309. 7689728

[42] Rittmaster RS, Manning AP, Wright AS, Thomas LN, Whitefield S, et al (1995) Evidence for atrophy and apoptosis in the ventral prostate of rats given the 5 alpha-reductase inhibitor finasteride. Endocrinology 136: 741–748. 7835306 10.1210/endo.136.2.7835306

[43] Gregory CW, Johnson RT Jr, Mohler JL, French FS, Wilson EM (2001) Androgen receptor stabilization in recurrent prostate cancer is associated with hypersensitivity to low androgen. Cancer Res 61: 2892–2898. 11306464

[44] Lee E, Salic A, Kruger R, Heinrich R, Kirschner MW (2003) The roles of APC and Axin derived from experimental and theoretical analysis of the Wnt pathway. PLoS Biol 1: E10. 14551908 10.1371/journal.pbio.0000010 PMC212691

[45] Wright AS, Thomas LN, Douglas RC, Lazier CB, Rittmaster RS (1996) Relative potency of testosterone and dihydrotestosterone in preventing atrophy and apoptosis in the prostate of the castrated rat. J Clin Invest 98: 2558–2563. 8958218 10.1172/JCI119074 PMC507713

[46] Barton HA, Andersen ME (1998) A model for pharmacokinetics and physiological feedback among hormones of the testicular-pituitary axis in adult male rats: a framework for evaluating effects of endocrine active compounds. Toxicol Sci 45: 174–187. 9848124 10.1006/toxs.1998.2538

[47] Attard G, Cooper CS, de Bono JS (2009) Steroid hormone receptors in prostate cancer: a hard habit to break? Cancer Cell 16: 458–462. 19962664 10.1016/j.ccr.2009.11.006

[48] Burd CJ, Petre CE, Morey LM, Wang Y, Revelo MP, et al (2006) Cyclin D1b variant influences prostate cancer growth through aberrant androgen receptor regulation. Proc Natl Acad Sci U S A 103: 2190–2195. 16461912 10.1073/pnas.0506281103 PMC1413684

[49] Gregory CW, He B, Johnson RT, Ford OH, Mohler JL, et al (2001) A mechanism for androgen receptor-mediated prostate cancer recurrence after androgen deprivation therapy. Cancer Res 61: 4315–4319. 11389051

[50] Petre-Draviam CE, Cook SL, Burd CJ, Marshall TW, Wetherill YB, et al (2003) Specificity of cyclin D1 for androgen receptor regulation. Cancer Res 63: 4903–4913. 12941814

[51] Sadar MD, Hussain M, Bruchovsky N (1999) Prostate cancer: molecular biology of early progression to androgen independence. Endocr Relat Cancer 6: 487–502. 10730903 10.1677/erc.0.0060487

[52] Jain HV, Clinton SK, Bhinder A, Friedman A (2011) Mathematical modeling of prostate cancer progression in response to androgen ablation therapy. Proc Natl Acad Sci U S A 108: 19701–19706. 22106268 10.1073/pnas.1115750108 PMC3241775

[53] Heinrich R, Schuster S (1996) The regulation of cellular systems. New York City: Chapman and Hall. 372 p.

[54] Fell D (1997) Understanding the control of metabolism. London: Portland Press. 300 p.

