# Supplementary material for: A Multiscale, Mechanism-Driven, Dynamic Model for the Effects of 5α-Reductase Inhibition on Prostate Maintenance
Source: PLoS One. 2012 Sep 6;7(9):e44359. doi: 10.1371/journal.pone.0044359 (PMC3435410; doi:10.1371/journal.pone.0044359)
Supplement: Figure S1 — FM captures kinetics dynamics of system following castration. (DOC) [file pone.0044359.s001.doc]

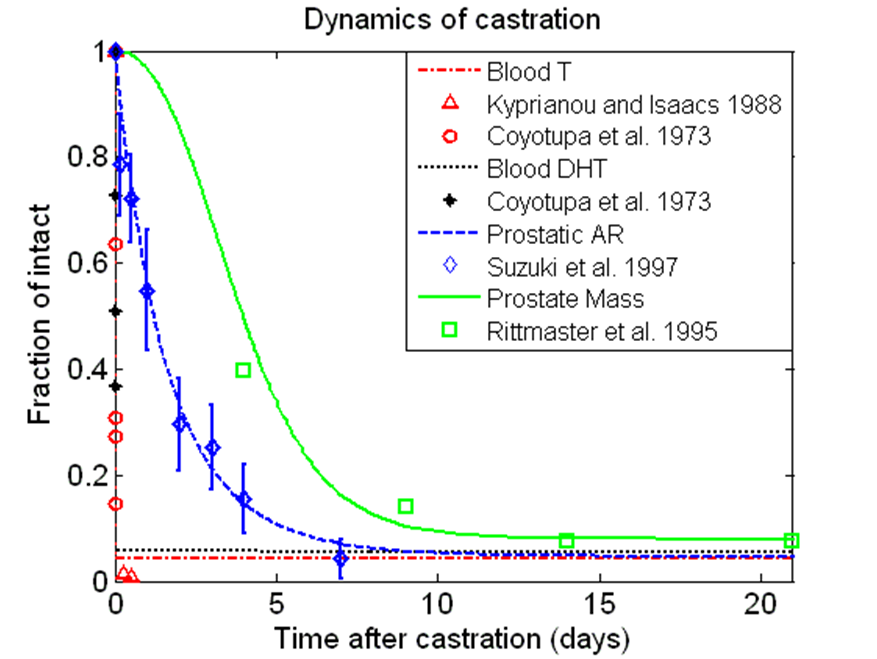


**Figure S1:** **FM captures kinetics dynamics of system following castration.** Following on Figure 10 of [36], this plot depicts system kinetics and dynamics immediately following castration in the rat. All lines represent model simulations. All simulated curves and data are normalized to unity. Within minutes, blood T and DHT levels significantly drop to near castrate levels. Within 5 days, prostatic AR concentrations are reduced to near castrate levels. Finally, after approximately 15 days, the prostate has regressed to less than 10% of its normal (intact) mass. FM has been specifically fit to the prostate mass data from the Rittmaster data set, so these are the only data depicted in this plot. Like PM, FM kinetics and dynamics remain in agreement with published data.
