# Supplementary material for: A Multiscale, Mechanism-Driven, Dynamic Model for the Effects of 5α-Reductase Inhibition on Prostate Maintenance
Source: PLoS One. 2012 Sep 6;7(9):e44359. doi: 10.1371/journal.pone.0044359 (PMC3435410; doi:10.1371/journal.pone.0044359)
Supplement: Table S1 — State variables for FM. (DOC) [file pone.0044359.s002.doc]

**Table S1: State variables for** FM

| Variable | Description | Units | Steady State |
| --- | --- | --- | --- |
| *AT_if_* | Amount of T in testicular interstitial fluid | nmol | 0.058 |
| *AT_st_* | Amount of T in testicular seminiferous tubules | nmol | 0.754 |
| *AT_b_* | Amount of T in brain/body | nmol | 0.103 |
| *AT_bl_* | Amount of T in blood | nmol | 0.108 |
| *AT_p_* | Amount of T in prostate | nmol | 7.23×10^-5^ |
| *AT_l_* | Amount of T in liver | nmol | 0.061 |
| *AD_l_* | Amount of DHT in liver | nmol | 0.045 |
| *AD_b_* | Amount of DHT in brain/body | nmol | 0.016 |
| *AD_bl_* | Amount of DHT in blood | nmol | 0.04 |
| *AD_p_* | Amount of DHT in prostate | nmol | 0.02 |
| *ALH* | Amount of LH in brain/body | nmol | 0.002 |
| *CT:A* | Concentration of T bound to albumin | nM | 4.71 |
| *CD:A* | Concentration of DHTT bound to albumin | nM | 1.82 |
| *CT:R* | Concentration of T:AR complex | nM | 0.061 |
| *CD:R* | Concentration of DHT:AR complex | nM | 14.5 |
| *CTT* | Concentration of T:AR-T:AR homodimer | nM | 0.0002 |
| *CDD* | Concentration of DHT:AR-DHT:AR homodimer | nM | 9.45 |
| *CDT* | Concentration of DHT:AR-T:AR heterodimer | nM | 0.04 |
| *CTT_cd_* | Concentration of T:AR-T:AR dimer bound to *cd* DNA | nM | 3.85×10^-7^ |
| *CTT_sec_* | Concentration of T:AR-T:AR dimer bound to *sec* DNA | nM | 3.6×10^-7^ |
| *CTT_cp_* | Concentration of T:AR-T:AR dimer bound to *cp* DNA | nM | 2.71×10^-7^ |
| *CTT_5a_* | Concentration of T:AR-T:AR dimer bound to *5aR2* DNA | nM | 9.83×10^-8^ |
| *CDD_cd_* | Concentration of DHT:AR-DHT:AR dimer bound to *cd* DNA | nM | 0.07 |
| *CDD_sec_* | Concentration of DHT:AR-DHT:AR dimer bound to *sec* DNA | nM | 0.065 |
| *CDD_cp_* | Concentration of DHT:AR-DHT:AR dimer bound to *cp* DNA | nM | 0.049 |
| *CDD_5a_* | Concentration of DHT:AR-DHT:AR dimer bound to *5aR2* DNA | nM | 0.018 |
| *CDT_cd_* | Concentration of T:AR-DHT:AR dimer bound to *cd* DNA | nM | 3.66×10^-5^ |
| *CDT_sec_* | Concentration of T:AR-DHT:AR dimer bound to *sec* DNA | nM | 3.42×10^-5^ |
| *CDT_cp_* | Concentration of T:AR-DHT:AR dimer bound to *cp* DNA | nM | 2.58×10^-5^ |
| *CDT_5a_* | Concentration of T:AR-DHT:AR dimer bound to *5aR2* DNA | nM | 9.35×10^-6^ |
| *AR* | Total amount of AR in prostate | nmol | 0.016 |
| *VPC_1_* | Volume/mass of androgen-sensitive prostatic cell mass^1^ | mg | 191 |
| *VPL_1_* | Volume/mass of androgen-sensitive prostatic ductal lumen mass^1^ | mg | 230 |
| A1 | Amount of finasteride in dosing compartment | nmol | 0 |
| A2 | Amount of finasteride in central compartment^2^ | nmol | 0 |
| A3 | Amount of finasteride in peripheral compartment | nmol | 0 |
| 5aR2 | Concentration of free 5aR2 enzyme in prostate | nM | 19 |
| 5aR2F | Concentration of 5aR2:finasteride complex in prostate | nM | 0 |
| 5aR2T | Concentration of 5aR2:T complex in prostate | nM | 1.0 |
| 5aR2F* | Concentration of time-dependent 5aR2:F complex in prostate^3^ | nM | 0 |

“Steady state” in the table heading referes to the value of the variable at steady state, which is equal to the initial value. Equations in the text denote amounts with *A* (e.g., *AT_if_* is amount of T in testicular

interstitial fluid). The initial values for these state variables can be obtained by dividing the steady state value for concentrations in the table (e.g., *CT_if_*) by the appropriate volume for the corresponding compartment. The reader is referred to [36] for calculations of all algebraic variables used in FM not already covered in the present manuscript.

^1^We assume 1 g = 1 mL tissue

^2^*A2* was calculated in nmol using the molecular weight of finasteride (372.55 g/mol). The algebraic variable *F* is calculated in nM from *A2* by using the central compartment volume of distribution (see [26]). The volume of distribution was normalized from the body weight used by Stuart and co-workers to the bodyweight (*bw*) used in FM.

^3^This complex is permanent in FM because the dissociation constant is zero (see main text).
