# Supplementary material for: A Multiscale, Mechanism-Driven, Dynamic Model for the Effects of 5α-Reductase Inhibition on Prostate Maintenance
Source: PLoS One. 2012 Sep 6;7(9):e44359. doi: 10.1371/journal.pone.0044359 (PMC3435410; doi:10.1371/journal.pone.0044359)
Supplement: Table S2 — Model parameters – physiological. (DOC) [file pone.0044359.s003.doc]

Table S1: Model parameters – physiological*

| Parameter | Description | Value | Reference |
| --- | --- | --- | --- |
| *bm* | Rat body mass | 0.3 kg | [36] |
| *V_bl_* | Volume of blood | 0.021 L | [36] |
| *V_l_* | Volume of liver | 0.012 L | [36] |
| *V_b_* | Volume of rest-of-body (ROB) excluding testes^1^ | 0.242 L | [36] |
| *V_bt_* | Volume of ROB including testes^1^ | 0.245 L | [36] |
| *V_t_* | Volume of testes | 0.0033 L | [36] |
| *V_it_* | Volume of interstitial tissue | 3.63 x 10^-4^ L | [36] |
| *V_st_* | Volume of seminiferous tubules | 0.0029 L | [36] |
| *V_if_* | Volume of interstitial fluid | 2.25 x 10^-4^ L | [36] |
| *VPL*_2_ | Basal prostatic ductal lumen volume^2^ | 18.2 mg | main text |
| *VPC*_2_ | Basal prostate cell volume^2^ | 18 mg | main text |
| *Q_c_* | Cardiac output | 6.08 L/hr | [36] |
| *Q_p0_* | Steady-state blood flow to prostate | 0.024 L/hr | [36] |
| *k_Qp_* | Proportional constant for *Q_p_*^3^ | 52.52 hr^-1^ | [36] |
| *Q_t_* | Blood flow rate to testes (intact only) | 0.061 L/hr | [36] |
| *Q_l_* | Blood flow rate to liver | 1.06 L/hr | [36] |
| *Q_b_* | Blood flow rate to ROB excluding testes | *Q_c_* - *Q_p_* - *Q_t_* - *Q_l_* | flux balance |
| *Q_bt_* | Blood flow rate to ROB including testes | *Q_c_* - *Q_pt_* - *Q_l_* | flux balance |
| *ts* | Fraction of testicular bloodflow shunted to SV | 0.56 | [36] |
| *CDNA_k_* | Total DNA concentration per site (*k* = *cp, cd, sec*) | 0.075 nM | [36] |
| *CDNA_5aR2_* | Total DNA concentration for 5aR2 gene | 0.019 nM | main text |
| *CA* | Total concentration of serum albumin | 5 x 10^5^ nM | [36] |
| *k_cp1_* | Prostate cell proliferation rate constant | 0.5 mg/hr | main text |
| *k_flo_* | Prostatic fluid production rate constant | 0.043 hr^-1^ | [36] |

*We assume 1 g = 1 mL tissue

^1^No body mass was reported in the Rittmaster data set [42], however the average intact prostate mass was reported to be approximately 457 g, signficantly larger than the original prostate mass used in PM (see [36]). Therefore, we increased the intact prostate mass in FM to match the Rittmaster data set and correspondingly decreased the volume of the ‘body’ compartment (see Figure 2 in the main text) so that the overal rat mass remains 0.3 kg.

^2^Changed from PM to match Rittmaster data set (see main text). There are no data in the Rittmaster data set that suggest the relative masses between androgen insensitive cellular and ductal lumen spaces. Since these are static masses in FM, they are easily adjusted, should appropriate data become available.

^3^This value changed from PM because the initial mass of the prostate in FM has changed from PM. This constant is determined by Q_p0_/V_p0_, where *V_p0_* is the steady-state mass (volume) of the prostate in FM. *V_p_* is calculated by adding the four distinct compartments for the prostate: *VPC_1_*, *VPL_1_*, *VPC_2_*, and *VPL_2_*.
