# Supplementary material for: A Multiscale, Mechanism-Driven, Dynamic Model for the Effects of 5α-Reductase Inhibition on Prostate Maintenance
Source: PLoS One. 2012 Sep 6;7(9):e44359. doi: 10.1371/journal.pone.0044359 (PMC3435410; doi:10.1371/journal.pone.0044359)
Supplement: Table S3 — Model parameters – Hormone pharmacokinetics and non-prostatic metabolism. (DOC) [file pone.0044359.s004.doc]

**Table S1: Model parameters – Hormone pharmacokinetics and non-prostatic metabolism**

| Parameter | Description | Value | Reference |
| --- | --- | --- | --- |
| *PT_if_* | IF/blood partition coefficient for T | 1.96 | [36] |
| *PT_st_* | ST/IF partition coefficient for T | 1.00 | [36] |
| *PT_l_* | Liver/blood partition coeff. for T | 2.75 | [36] |
| *PD_l_* | Liver/blood partition coeff. for DHT | 2.0 | [36] |
| *PT_b_* | ROB/blood partition coeff. for T | 1.00 | [36] |
| *PD_b_* | ROB/blood partition coeff. for DHT | 0.62 | [36] |
| *μ* | Testicular permeability coefficient | 1000 L/hr | [36] |
| *NT_p_* | Coeff. for non-specific T binding in prostate | 0.79 | [36] |
| *ND_p_* | Coeff. for non-specific DHT binding in prostate | 1.00 | [36] |
| *k_TAon_* | Association rate for T-albumin | 0.18 nM^-1^ hr^-1^ | [36] |
| *k_TAoff_* | Dissociation rate for T-albumin^1^ | 8100 hr^-1^ | [36] |
| *k_DAon_* | Association rate for DHT-albumin | 0.15 nM^-1^ hr^-1^ | [36] |
| *k_DAoff_* | Dissociation rate for DHT-albumin^1^ | 4335 hr^-1^ | [36] |
| *α_T_* | Relative potency of T for LH synthesis inhibition | 0.25 | [36] |
| *α_D_* | Relative potency of DHT for LH synthesis inhibition | 0.75 | [36] |
| *k*_1_*_t_* | T synthesis rate in testes (intact only) | 40.32 L/hr | [36] |
| *k_2_*_t_ | Basal T synthesis rate | 0.17 nmol/hr | [36] |
| *k_lT_* | T elimination rate in liver | 87.93 hr^-1^ | [36] |
| *k_lD_* | DHT elimination rate in liver | 77.2 hr^-1^ | [36] |
| *k_LH1_* | T inhibition constant 1 for LH | 0.13 L/hr | [36] |
| *k_LH2_* | T inhibition constant 2 for LH | 0.026 nmol/hr | [36] |
| *k_LH3_* | Minimum LH synthesis rate | 1.68 x 10^-4^ nmol/hr | [36] |
| *k_LH4_* | LH degradation rate constant | 0.8 hr^-1^ | [36] |
| *V_maxl_* | *V_max_* for T metabolism to DHT in liver | 3.65 nmol/hr | [36] |
| *k_m5a_* | *k_m_* for T metabolism to DHT in liver | 2.3 nM | [26] |
| *K_i_^5aR1^* | 5aR1 inhibition constant (K_i_) for finasteride | 5.4 nM | [26] |

^1^Slight difference in value from PM due to numerical truncations.
