# Supplementary material for: A Multiscale, Mechanism-Driven, Dynamic Model for the Effects of 5α-Reductase Inhibition on Prostate Maintenance
Source: PLoS One. 2012 Sep 6;7(9):e44359. doi: 10.1371/journal.pone.0044359 (PMC3435410; doi:10.1371/journal.pone.0044359)
Supplement: Table S4 — Model parameters – Prostate biochemistry and metabolism. (DOC) [file pone.0044359.s005.doc]

Table S1: Model parameters – Prostate biochemistry and metabolism

| Parameter | Description | Value | Reference |
| --- | --- | --- | --- |
| *k*_1_*_R_* | Basal AR synthesis rate | 68.15 nmol/hr | [36] |
| *ke_R_* | AR elimination rate | 71.9 L/hr | [36] |
| *k_TRon_* | Association rate for T-AR | 0.14 nM^-1^ hr^-1^ | [36] |
| *k_TRoff_* | Dissociation rate for T-AR | 0.069 hr^-1^ | [36] |
| *k_DRon_* | Association rate for DHT-AR | 0.053 nM^-1^ hr^-1^ | [36] |
| *k_DRoff_* | Dissociation rate for DHT-AR | 0.018 hr^-1^ | [36] |
| *k_IJon_* | Association rates for androgen dimerization (I and J represent the two androgens in the dimer) | 0.14 nM^-1^ hr^-1^ | [36] |
| *k_IJoff_* | Dissociation rates for androgen dimerization | 3.13 hr^-1^ | [36] |
| *k_DNAonIJk_* | Association rates for DNA-dimer formation (I and J represent the two dimers and k denotes the representative genes (*cd, cp, 5a, sec*) | 0.14 nM^-1^ hr^-1^ | [36] |
| *k_DNAoffIJk_* | Dissociation rates for DNA-dimer formation^1^ | see footnote | [36] |
| *k_1_* | Association rate constant for T:5aR2^2^ | 500 hr^-1^nM^-1^ | main text |
| *k_2_* | Dissociation rate constant for T:5aR2 | 30 hr^-1^ | main text |
| *k_cat_* | Catalysis rate constant for T 🡪 DHT | 270 hr^-1^ | [39] |
| *K_m_* | K_m_ for T 🡪 DHT | 0.6 nM | [26] |
| *k_3_* | Association rate constant for finasteride:5aR2^2^ | 1000 hr^-1^nM^-1^ | main text |
| *k_4_* | Dissociation rate constant for finasteride:5aR2 | 500 hr^-1^ | main text |
| *k_5_* | Rate constant for *5aR2F* 🡪 *5aR2F** | 3.96 hr^-1^ | [26] |
| *k_6_* | Rate constant for *5aR2F** 🡪 *5aR2F* | 0 hr^-1^ | [26] |
| *K_i_^5aR2^* | K_i_ for finasteride:5aR2 | 0.5 nM | [26] |
| *k_dg_* | Degradation rate constant for 5aR2^2^ | 0.016 hr^-1^ | fitted |

^1^For a description on calculation of these values, see [36] and the main text. Note that a key parameter in these calculations, *k_DNAoffTT_*, has changed in FM to 1.6 (see Table 7 in the main text).

^2^Also see Table 4 of main text.
