# Supplementary material for: A Multiscale, Mechanism-Driven, Dynamic Model for the Effects of 5α-Reductase Inhibition on Prostate Maintenance
Source: PLoS One. 2012 Sep 6;7(9):e44359. doi: 10.1371/journal.pone.0044359 (PMC3435410; doi:10.1371/journal.pone.0044359)
Supplement: Table S5 — Model parameters – Finasteride pharmacokinetic (PK) model. (DOC) [file pone.0044359.s006.doc]

Table S5: Model parameters – Finasteride pharmacokinetic (PK) model*

| Parameter | Description | Value | Reference |
| --- | --- | --- | --- |
| *V*_c_ | Volume of central compartment^1^ | 0.54 L | [26] |
| *k_a_* | Absorption rate constant | 0.931 hr^-1^ | [26] |
| *k_23_* | Inter-compartment transfer | 0.161 hr^-1^ | [26] |
| *k_32_* | Inter-compartment transfer | 0.254 hr^-1^ | [26] |
| *k_20_* | Elimination rate constant | 0.965 hr^-1^ | [26] |

*See Figure 3 of the main text

^1^Value normalized by body mass from Stuart value to FM value: 0.5(bm/0.28).
