# Supplementary material for: A Multiscale, Mechanism-Driven, Dynamic Model for the Effects of 5α-Reductase Inhibition on Prostate Maintenance
Source: PLoS One. 2012 Sep 6;7(9):e44359. doi: 10.1371/journal.pone.0044359 (PMC3435410; doi:10.1371/journal.pone.0044359)
Supplement: Text S1 — Calculation of the algebraic variables for gene occupancy in FM. (DOC) [file pone.0044359.s007.doc]

**Text S1: Calculation of the algebraic variables for gene occupancy in FM**

The algebraic expressions to calculate the gene occupancies for each of the four genes in FM (*DNAo*) remain unchanged from PM. These expressions are well characterized in [36], under “prostate compartment” in the methods section. For a detailed description, the reader is referred to that publication. Briefly, gene occupancies are denoted by *DNAo_i_*, where *i =* anti-apoptosis or cell death (*cd*), fluid secretion (*sec*), cell proliferation (*cp*), or 5aR2 production (*5aR*). Each gene occupancy expression is calculated by dividing the total concentration of DNA-bound androgen:AR dimers (including T and DHT homodimers as well as T-D heterodimers – see Figure 1 of the main article) by the total number of DNA binding sites for that gene (given in concentration). The concentration for each DNA-bound androgen:AR dimer is determined through an ordinary differential equation with standard binding kinetic terms governed by free DNA sites, occupied DNA sites, and the respective *k_on_* and *k_off_* values (see Equations 16-26 in [36]).
