# Supplementary material for: A Multiscale, Mechanism-Driven, Dynamic Model for the Effects of 5α-Reductase Inhibition on Prostate Maintenance
Source: PLoS One. 2012 Sep 6;7(9):e44359. doi: 10.1371/journal.pone.0044359 (PMC3435410; doi:10.1371/journal.pone.0044359)
Supplement: Text S2 — Forced steady state on prostate cell growth and death dynamics. (DOC) [file pone.0044359.s008.doc]

**Text S2: Forced steady state on prostate cell growth and death dynamics**

To force steady state on Equation 10 from the main article for androgen-sensitive prostate cellular mass *VPC_1_* at the initial value (*VPC_1b_*), we derived the following relationship between the cell growth parameter (*k_cp1_*) and cell death parameter (*k_cd1_*):

$$k_{cd1}=k_{cp1}\frac{DNAo_{cp0}}{VPC_{1b}\left(1-DNAo_{cd0}\right)},$$

where DNAo_cp0_ and DNAo_cd0_ are the initial (steady state) values for the cell proliferation and anti-apoptosis genes, respectively. Then, at steady state (intact condition), the right-hand side of the equation for *VPC_1_* becomes:

$$k_{cp1}DNAo_{cp}-k_{cp1}\frac{DNAo_{cp0}}{VPC_{1b}\left(1-DNAo_{cd0}\right)}\left(1-DNAo_{cd}\right)VPC_{1}=0$$

thus enforcing the steady state value of *VPC_1b_*.
