# Supplementary material for: A Multiscale, Mechanism-Driven, Dynamic Model for the Effects of 5α-Reductase Inhibition on Prostate Maintenance
Source: PLoS One. 2012 Sep 6;7(9):e44359. doi: 10.1371/journal.pone.0044359 (PMC3435410; doi:10.1371/journal.pone.0044359)
Supplement: Text S3 — Differential Equations for FM. (DOC) [file pone.0044359.s009.doc]

**Text S3: Differential Equations for FM***

$\begin{matrix} \frac{dA1}{dt}=-k_{a}A1 \\ {}^{5}\frac{dA2}{dt}=k_{a}A1-k_{23}A2+k_{32}A3-k_{20}A2+k_{4}5aR2F-k_{3}5aR2\cdot F \\ \frac{dA3}{dt}=k_{23}A2-k_{32}A3 \\ \frac{d5aR2}{dt}=-k_{3}5aR2\cdot F+k_{4}5aR2F-k_{1}5aR2\cdot T+k_{2}5aR2T+5aR2syn-5aR2dg+k_{cat}5aR2T \\ \frac{d5aR2F}{dt}=-k_{4}5aR2F+k_{3}5aR2\cdot F-k_{5}5aR2F+k_{6}5aR2F^{*} \\ \frac{d5aR2T}{dt}=-k_{2}5aR2T+k_{1}5aR2\cdot T-k_{cat}5aR2T \\ \frac{d5aR2F^{*}}{dt}=-k_{6}5aR2F^{*}+k_{5}5aR2F \end{matrix}$

*All algebraic variables in FM derived from these state variables are described in the main text, supporting infromation and in [36].

^1^The equation for CD:A is similarly derived.

^2^The equation for CD:R is similarly derived.

^3^The equations for CDD and CTT are similarly derived.

^4^The equations for all 12 DNA-bound dimers (see Table S1 and [36]) are similarly derived.

^5^The equation for the central compartment in [26] has been modified in FM to account for binding to prostatic 5aR2.
